# Active earth pressure against flexible retaining wall for finite soils under the drum deformation mode

**DOI:** 10.1038/s41598-021-04411-4

**Published:** 2022-01-11

**Authors:** Weidong Hu, Xinnian Zhu, Yongqing Zeng, Xiaohong Liu, Chucai Peng

**Affiliations:** College of Civil Engineering and Architecture, Hunan Institute of Science and Technology, Yueyang, 414000 China

**Keywords:** Civil engineering, Natural hazards

## Abstract

A reasonable method is proposed to calculate the active earth pressure of finite soils based on the drum deformation mode of the flexible retaining wall close to the basement’s outer wall. The flexible retaining wall with cohesionless sand is studied, and the ultimate failure angle of finite soils close to the basement’s outer wall is obtained using the Coulomb theory. Soil arch theory is led to get the earth pressure coefficient in the subarea using the trace line of minor principal stress of circular arc after stress deflection. The soil layers at the top and bottom part of the retaining wall are restrained when the drum deformation occurs, and the soil layers are in a non-limit state. The linear relationship between the wall movement’s magnitude and the mobilization of the internal friction angle and the wall friction anger is presented. The level layer analysis method is modified to propose the resultant force of active earth pressure, the action point’s height, and the pressure distribution. Model tests are carried out to emulate the process of drum deformation and soil rupture with limited width. Through image analysis, it is found that the failure angle of soil within the limited width is larger than that of infinite soil. With the increase of the aspect ratio, the failure angle gradually reduces and tends to be constant. Compared with the test results, it is shown that the horizontal earth pressure reduces with the reduction of the aspect ratio within critical width, and the resultant force decreases with the increase of the limit state region under the same ratio. The middle part of the distribution curve is concave. The active earth pressure strength decreases less than Coulomb’s value, the upper and lower soil layers are in the non-limit state, and the active earth pressure strength is more than Coulomb’s value.

## Introduction

Deep foundation pits are often excavated near the basement of existing buildings in urban and municipal engineering. The undisturbed soil between the retaining wall and the existing wall is narrow, and its width is limited, which is also the research object of this paper. Row pile wall, underground diaphragm wall, and sheet pile wall have been extensively used in enclosure structure of foundation pit engineering and slope engineering. The thickness of the retaining wall structure is minimal compared with the height. The wall has obvious flexure deformation, which cannot meet the assumption regarding the rigid retaining wall, called the flexible retaining wall. The classical earth pressure theories of Coulomb and Rankine cannot accurately predict the earth pressure on the flexible retaining wall.

The structural deformation of the retaining wall caused by the excavation of internal support and anchor pull system can be classified into three types^1–3^. The first type is a cantilevered triangle with the most significant displacement at the top of the wall. The second type is drum deformation because the upper part of the flexible retaining wall is supported. The wall is embedded in the soil, indicating that the displacement of the top and bottom remains unchanged. The abdomen of the retaining wall structure protrudes into the foundation pit, and the displacement curve is parabolic. The third type of deformation is the combination of the first two. For the supporting and anchoring flexible retaining wall, the drum deformation mode bulging into the pit is the most typical one, which is also the basis for studying other combined wall movement modes. It is of great significance to study the deformation’s behavior, the failure mechanism, and the earth pressure distribution.

The drum deformation mode of the flexible retaining wall is characterized by large deformation in the middle and small deformation at both ends. The horizontal displacement of soil is mostly parabolic. The earth pressure on the retaining wall is nonlinear along with the wall’s height, which is affected by the magnitudes of displacement and the displacement mode of wall movement. Milligan^2^ carried out the model test of flexible retaining wall with support at the top, studied the relationship between the drum deformation of the wall and the displacement of the soil, and the development of sliding surface behind the wall. Lu et al.^4^ carried out the active earth pressure and displacement tests of a cantilever and single anchor flexible retaining wall. They obtained the R-shaped distribution of active earth pressure along the anchored retaining wall. Zhang et al.^3^ presented the relationship between the coefficient of earth pressure of sand and the increment ratio of axial and lateral strain based on the triaxial test. They deduced the unified expression of displacement and the calculation method of earth pressure under any displacement state. Based on the previous experiments and numerical analysis, the calculation method of active earth pressure resultant force and its distribution on flexible retaining walls under arbitrary displacement is proposed by Ying et al.^5^. However, the above earth pressure research does not involve the retaining wall adjacent to the outer wall or vertical slope and the soil with limited width.

Under the drum deformation mode, the wall's top and bottom are constrained by the support and the soil layers, respectively. The deformation feature can be seen as the upper wall rotates outward around the top of the wall while the lower wall revolves outward around the bottom of the wall^6–10^. There is a relative displacement tendency between the upper and lower soils during the deformation, resulting in the horizontal shearing stress, which cannot be ignored. Therefore, the coefficient and distribution of active earth pressure are affected. As a result, the soil layer's deformation and earth pressure distribution near the top and bottom of the wall have RT mode and RB mode characteristics.

The existing theoretical research is still insufficient. Based on the relationship between the unit earth pressure and the horizontal displacement, the calculation formulas^3,11,12^ were put forward, but the relative displacement was not considered under the drum deformation. The results show that earth pressure distribution is always between the static and active states, which can’t reflect the redistribution of earth pressure caused by the drum deformation of flexible retaining walls. Ying et al.^13^ considered the relative displacement of the adjacent depth soil layers, but the earth pressure dropped sharply at the wall’s maximum displacement, which was unreasonable.

The deformation of the soil layer near the top and bottom of the retaining wall is limited, and it is impossible to reach a limit state in company with the soil layer in the middle abdomen. The rotating angle of the retaining wall is slight when in service, which makes the displacement of the soil near the top and bottom of the wall very small, and it is not easy to reach the limit state. As a result, the soil shearing strength and friction between wall and soil can’t be fully mobilized, and they are actually in a non-limit active state. The magnitude of active earth pressure is affected by the drum deformation mode, which results in the redistribution of earth pressure. The soil layer’s active earth pressure near the top and bottom of the wall increases due to the soil arching^14–16^, while the active earth pressure of the soil layer in the middle of the wall decreases relatively.

Fathipour et al.^17,18^ analyzed the lateral earth pressures exerted on retaining walls having an unsaturated backfill and backfilled with geosynthetic-reinforced soil strata with finite element limit analysis using second-order cone programming. Fathipour et al.^19,20^ evaluated the modified pseudo-dynamic lateral earth pressures acting on retaining structure filled with granular backfill and filled with an anisotropic medium of dry and noncohesive material. Mirmoazen et al.^21^ conducted a detailed numerical study to evaluate the lateral earth pressure acting on geosynthetic-reinforced retaining walls with an anisotropic granular backfill subjected to strip footing loadings. In the above research, the finite element lower limit analysis coupled with second-order cone programming is introduced into the retaining wall structure stability analysis and earth pressure calculation. However, the non-limit state of soil and soil arching effect is not considered in the earth pressure calculation.

Research on lateral earth pressures exerted on flexible retaining walls with limited granular soil^22–25^ has attracted more and more attention in practical engineering. Therefore, to better study the active earth pressure against the flexible retaining wall, the wall movement mode, the actual non-limit active state of the soil layer near the top and bottom of the wall, and the soil arching should be taken into account^16,26–30^.

The soil arch theory is led into this research based on the progressive rupture mechanism in the cohesionless sand under drum formation mode. First, the differential level layer method is applied to analyze the partition unit. Then, considering the shearing stress and the partial mobilization of shearing strength and wall friction in a non-limit state, the distribution of the active earth pressure, the resultant force’s magnitude, and the action point’s height are obtained. Finally, the model tests are conducted to verify further the proposed method in the paper.

## Analysis model of flexible retaining wall

**Figure 1:**
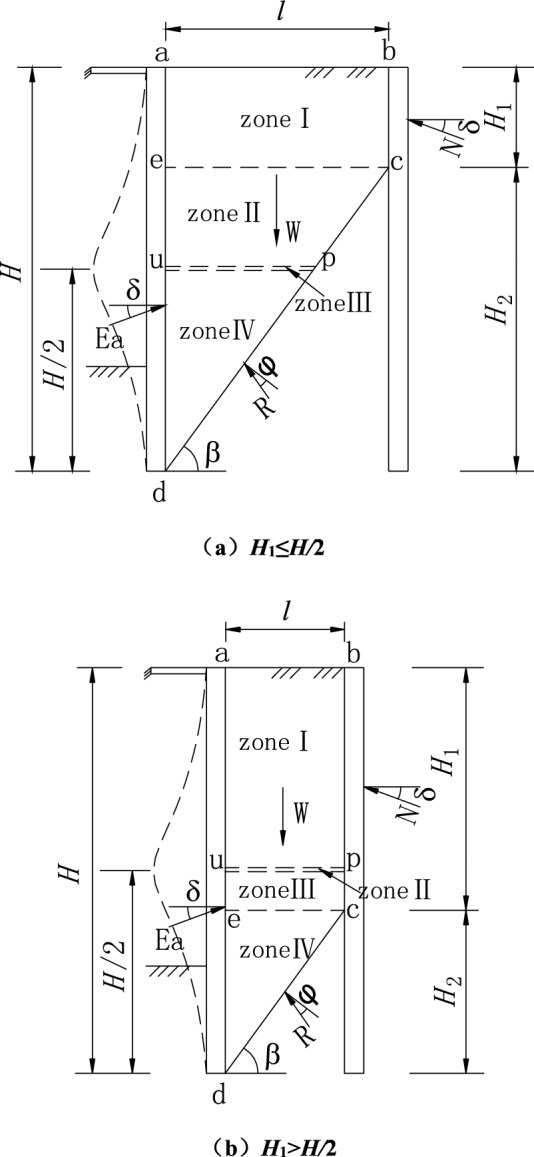
Slip surface.

The retaining wall is close to the outer basement wall or vertical rock slope, and the height of the retaining wall is *H*, as shown in Fig. 1. Cohesionless sand is filled behind the retaining wall, and the narrow width is *l* = *n∙H* (*n* is the ratio of width to height).

The middle abdomen of the flexible retaining wall protrudes into the excavation under the top strut’s support and the constraint of the embedded end at the bottom, forming a drum deformation. The only rotation occurs at the top and bottom of the retaining wall, and its horizontal displacement is assumed to be zero. It is assumed that the midpoint *H/*2 is the place of maximum deformation and horizontal displacement. Because of the limited width of retaining sand, the slip plane is cut off by the outer wall or rock slope and cannot fully develop to the sand top surface. Thus, the height of the wall is divided into *H*_1_ and *H*_2_. The sliding surface is assumed to be a plane, passing through the bottom of the wall and forming an angle *β* with the level plane.

Due to the different widths of the limited soil, the intersection point *c* of slip surface and vertical external wall or rock slope may be higher or lower than the maximum horizontal displacement of the midpoint, including two cases, as shown in Fig. 1. When *H*_1_ ≤ *H/*2, the soil mass is divided into upper and lower areas with *ce* as the boundary. The area above the *ce* boundary is zone I, and the area below the *ce* boundary is divided into zones II, III, and IV from top to bottom. The *up* thin layer at the maximum horizontal displacement is the intermediate transition zone III (Fig. 1a). When the width is minimal, *H*_1_ > *H/*2, the area below the *ce* boundary is zone IV, and the area above the *ce* boundary is divided into zones I, II, and III from top to bottom. The *up* thin layer at the maximum horizontal displacement is the intermediate transition zone II (Fig. 1b).

Based on the wall friction and the existence of force *N*, for simplifying the calculation, it is approximately considered that the earth pressure distribution on the wall meets the triangular distribution along with the height. Therefore, it is the same at the same depth^16,31,32^. By introducing the parameter *m*, then:

1 $$N = mE_{a} = \left( {\frac{{H_{1} }}{H}} \right)^{2} E_{a} .$$

*E*_*a*_ is the resultant force of active earth pressure acting below the normal, and its direction is *δ* angle from the normal of the back of the wall.

Based on the Coulomb method, the vertical and horizontal equilibrium function on the sliding surface is derived.

2 $$E_{a} = \frac{{\frac{1}{2}\gamma nH^{2} (2 - n\tan \beta )}}{{\sin \delta [1 + (1 - n\tan \beta )^{2} ] + \cos \delta \cot (\beta - \varphi )[1 - (1 - n\tan \beta )^{2} ]}}.$$

The extreme value of *E*_a_ can be solved ($dE_{a} /d\beta = 0$) to obtain the value of *β* of the most dangerous sliding surface as the soil enters an active limit state. The value of the extreme thrust *E*_*a*_ can be obtained by using Eq. (2)^16^.

## The active earth pressure coefficient

Firstly, taking the situation shown in Fig. 1a as the research object, the earth pressure coefficient and the soil arching are analyzed. *ce* is taken as the boundary line according to the different boundary conditions of the finite soil.

**Figure 2:**
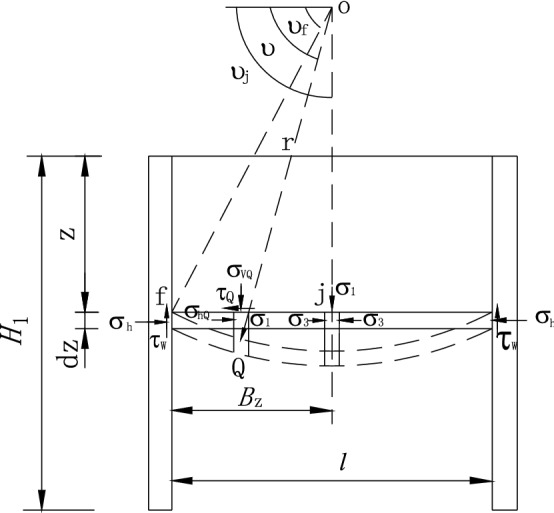
Trajectory of minor principal stress of zone I.

**Figure 3:**
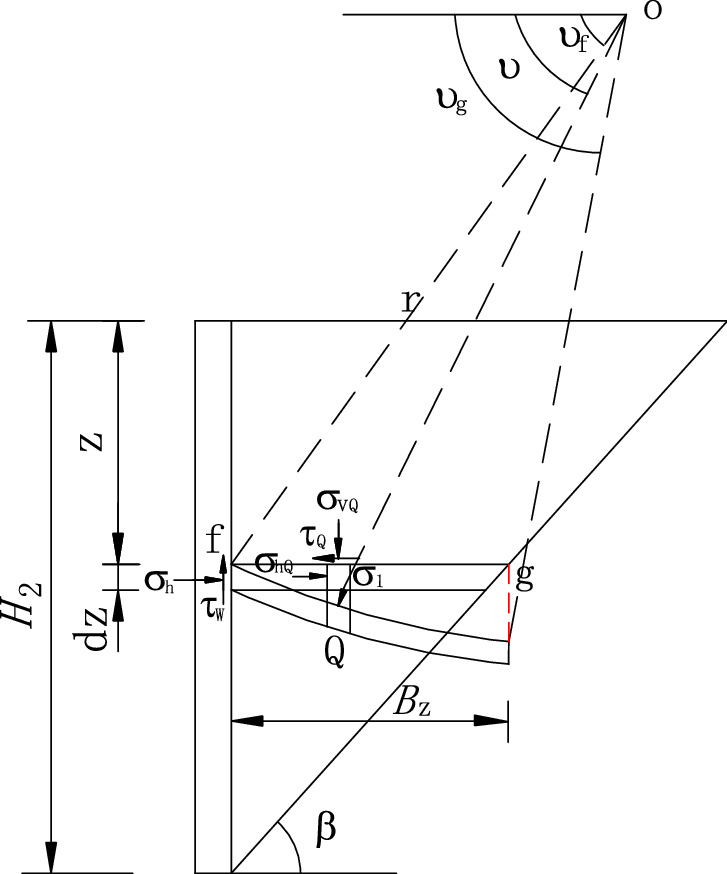
Trajectory of minor principal stress in zone I, III, IV.

The upper zone I is located between the backs of the retaining wall and the outer wall. In the process of drum deformation and ground subsidence, because of friction between two vertical parallel walls, the stress deflection occurs due to the soil arch, and the horizontal stress on the retaining wall is no longer minor principal stress. Therefore, the horizontal layer unit at depth *z* (in zone I) is taken for analysis. Each point's minor principal stress trajectories are connected to form a continuous arch curve, as shown in Fig. 2. Although the stress and boundary conditions of the retaining soil in the lower zones II, III, and IV are different from those in the upper zone I, the vertical and lateral deformation are also limited by frictions (the interface friction between the retaining wall and the soil and the soil friction on the failure plane). Therefore, the direction of the principal stress deflects, and its magnitude remains unchanged along the arch. Taking the layer unit at depth *z* (in zones II, III, and IV) for analysis shown in Fig. 3, each point’s minor principal stress trajectories on the horizontal unit form a half arch. Moreover, the horizontal shearing stress exists on the level unit’s surface objectively, and the shearing stress of each point is not equal because of the unequal deflection angle of each point.

The circular arch is generally employed for analysis by many scholars^16,26,33,34^. Since the circular arch’s calculation results are close to those of other shapes of arch curves, the circular arch stress trajectory is used to establish the calculation model in this paper.

After stress deflection occurs, an arched curve with radius *r* is formed at point *f*. The center of the circle is point *O*. The vertical stress distribution on the layer unit at depth *z* is uneven considering the soil arching effect. Herein, according to the study by Handy^15^ and Paik and Salgado^14^, the lateral active earth pressure coefficient *K*_awn_ is defined as

3 $$K_{awn} = \frac{{\sigma_{h} }}{{\overline{{\sigma_{v} }} }},$$

where $\sigma_{h}$ is the normal earth pressure on the interface between retaining wall and soil at the depth *z*, $\overline{{\sigma_{v} }}$ is the average erect pressure on the level at the same height.

The stress of point *Q* on the arch line is expressed as follows.

4 $$\left. \begin{gathered} \sigma_{vQ} =\frac{{\sigma_{1} }}{1 + \sin \varphi }(1 - \sin \varphi \cos 2\theta ) \hfill \\ \sigma_{hQ} =\frac{{\sigma_{1} }}{1 + \sin \varphi }(1 + \sin \varphi \cos 2\theta ) \hfill \\ \tau_{Q} =\frac{{\sigma_{1} \sin \varphi }}{1 + \sin \varphi }\sin 2\theta \hfill \\ \end{gathered} \right\},$$

where $\sigma_{1}$ is the major principal stress and *θ* is the deflection angle between the major principal stress and the level at point *Q*. The deflection angles at point *f* and *g* are indicated as:

5 $$\left. \begin{gathered} \theta_{f} = [\pi - \arcsin \left( {\frac{\sin \delta }{{\sin \varphi }}} \right) + \delta ]/2 \hfill \\ \theta_{g} = \pi /4 - \varphi /2 + \beta \hfill \\ \end{gathered} \right\}.$$

Considering the symmetry of soil mass in zone I, half of the circular arch trajectory can be taken for analysis. The horizontal span *B*_z_ is *l/*2, the deflection angle at point *j* is *θ*_*j*_ = π/2, and the average vertical stress along the arch in zone I is

6 $$\overline{{\sigma_{v} }} = \int_{{\theta_{f} }}^{{\theta_{j} }} {\frac{{\sigma_{vQ} r\sin \theta d\theta }}{{B_{z} }}} = \sigma_{1} - \frac{{2\sin \varphi \cos^{2} \theta_{f} }}{3(1 + \sin \varphi )}\sigma_{1} .$$

In the formula, the curve radius of the minor principal stress arch curve is *r* $= B_{z} /(\cos \theta_{f} - \cos \theta_{j} )$. The lateral coefficient of active earth pressure *K*_*awn*1_ in zone I can be presented from Eqs. (4) and (6),

7 $$K_{awn1} = \frac{{\sigma_{h} }}{{\overline{{\sigma_{v} }} }} = \frac{{3(1 + \sin \varphi \cos 2\theta_{f} )}}{{3(1 + \sin \varphi ) - 2\sin \varphi \cos^{2} \theta_{f} }}.$$

The mean shearing stress of soil arching line in zone I is

8 $$\overline{\tau } = \int_{{\theta_{f} }}^{{\theta_{j} }} {\frac{{\tau_{Q} r\sin \theta d\theta }}{{B_{z} }}} = \frac{{2\sin \varphi (1 - \sin^{3} \theta_{f} )}}{{3(1 + \sin \varphi )\cos \theta_{f} }}\sigma_{1} .$$

The average shearing stress coefficient *k* is defined as average shearing stress ratio to average vertical stress on the level layer unit, which should be less than *tanφ*.

9 $$k = \frac{{\overline{\tau } }}{{\overline{{\sigma_{v} }} }}.$$

Thus, the average shearing stress coefficient *k*_1_ of soil in zone I can be given

10 $$k_{1} = \frac{{\overline{\tau } }}{{\overline{{\sigma_{v} }} }} = \frac{{2\sin \varphi (1 - \sin^{3} \theta_{f} )}}{{3(1 + \sin \varphi )\cos \theta_{f} - 2\sin \varphi \cos^{3} \theta_{f} }}.$$

To the lower soil in zones II, III and IV, the average vertical stress on the track line of minor principal stress arching can be expressed as

11 $$\overline{{\sigma_{v} }} = \int_{{\theta_{f} }}^{{\theta_{g} }} {\frac{{\sigma_{vQ} r\sin \theta d\theta }}{{B_{z} }}} = \sigma_{1} - \frac{{2\sin \varphi (\cos^{3} \theta_{f} - \cos^{3} \theta_{g} )}}{{3(1 + \sin \varphi )(\cos \theta_{f} - \cos \theta_{g} )}}\sigma_{1} ,$$

where, the radius *r* $= B_{z} /(\cos \theta_{f} - \cos \theta_{g} )$. Similarly, the lateral coefficient of active earth pressure *K*_*awn*2_ = *K*_*awn*3_ = *K*_*awn4*_ and the coefficient of average shearing stress *k*_2_ = *k*_3_ = *k*_4_ can be obtained in zones II, III and IV

12 $$K_{awn2} = \frac{{\sigma_{h} }}{{\overline{{\sigma_{v} }} }} = \frac{{3(1 + \sin \varphi \cos 2\theta_{f} )}}{{3(1 + \sin \varphi ) - 2\sin \varphi (\cos^{2} \theta_{f} + \cos^{2} \theta_{g} + \cos \theta_{f} \cos \theta_{g} )}},$$

13 $$k_{2} = \frac{{\overline{\tau } }}{{\overline{{\sigma_{v} }} }} = \frac{{2\sin \varphi (\sin^{3} \theta_{g} - \sin^{3} \theta_{f} )}}{{3(1 + \sin \varphi )(\cos \theta_{f} - \cos \theta_{g} ) - 2\sin \varphi (\cos^{3} \theta_{f} - \cos^{3} \theta_{g} )}}.$$

Suppose the ultimate rupture angle is *β* = π/4 + *φ*/2, *K*_*awn*1_ = *K*_*awn*2_, *k*_1_ = *k*_2_. If *β* = *π*/4 + *φ*/2 and *δ* = 0, Eqs. (7) and (12) is able to transform into *K*_*awn*1,2_ = tan^2^(*π*/4 − *φ*/2), that is Rankine coefficient.

Furthermore, taking the situation shown in Fig. 1b as the research object, the coefficient of earth pressure and soil arching are analyzed according to the same method above. Then, we can get the lateral active earth pressure coefficients *K*_*awn*1_, *K*_*awn*2_, *K*_*awn*3_, and average shearing stress coefficients *k*_1_, *k*_2_, *k*_3_ in zones I, II, and III.

14 $$K_{awn1} = K_{awn2} = K_{awn3} = \frac{{3(1 + \sin \varphi \cos 2\theta_{f} )}}{{3(1 + \sin \varphi ) - 2\sin \varphi \cos^{2} \theta_{f} }},$$

15 $$k_{1} = k_{2} = k_{3} = \frac{{2\sin \varphi (1 - \sin^{3} \theta_{f} )}}{{3(1 + \sin \varphi )\cos \theta_{f} - 2\sin \varphi \cos^{3} \theta_{f} }}.$$

The coefficient of lateral active earth pressure Kawn4 and average shearing stress k4 in zone IV are obtained.

16 $$K_{awn4} = \frac{{3(1 + \sin \varphi \cos 2\theta_{f} )}}{{3(1 + \sin \varphi ) - 2\sin \varphi (\cos^{2} \theta_{f} + \cos^{2} \theta_{g} + \cos \theta_{f} \cos \theta_{g} )}},$$

17 $$k_{4} = \frac{{2\sin \varphi (\sin^{3} \theta_{g} - \sin^{3} \theta_{f} )}}{{3(1 + \sin \varphi )(\cos \theta_{f} - \cos \theta_{g} ) - 2\sin \varphi (\cos^{3} \theta_{f} - \cos^{3} \theta_{g} )}}.$$

## Parameter value in non-limit state

The horizontal displacement of the retaining wall is *s* under the drum movement mode, and the magnitude of displacement at the middle point is the largest, which value is *s*_max_. Assuming that the horizontal displacement required for the soil to enter the full limit state is *s*_a_, the area with the displacement *s* ≥ *s*_a_ is the entire limit state area, where the internal friction angle of the fill and the external wall friction angle are fully mobilized. In this paper, the region is defined as the intermediate transition region.

The area with horizontal displacement *s* < *s*_a_ of retaining wall is a non-limit state area. In the non-limit state, because of the small magnitude of displacement, the soil friction angle *φ*′ and the wall friction angle *δ*′ partial mobilize that their values are between the initial state values *φ*_0_, *δ*_0_, and the ultimate state values *φ*, *δ*, respectively. Considering that the mobilization of *φ*′ and *δ*′ are affected by the magnitude of horizontal displacement of the retaining wall, it is assumed that *φ*′ and *δ*′ increase linearly with the rise of horizontal displacement, the following expression is given by

18 $$\left. \begin{gathered} 0 \le z \le \frac{H}{2} - \frac{\Delta z}{2},\varphi ^{\prime} = \tan^{ - 1} [\tan \varphi_{0} + \frac{z}{H/2 - \Delta z/2}(\tan \varphi - \tan \varphi_{0} )] \hfill \\ \frac{H}{2} - \frac{\Delta z}{2} \le z \le \frac{H}{2} + \frac{\Delta z}{2},\varphi ^{\prime} = \varphi \hfill \\ \frac{H}{2} + \frac{\Delta z}{2} \le z \le H,\varphi ^{\prime} = \tan^{ - 1} [\tan \varphi_{0} + \frac{H - z}{{H/2 - \Delta z/2}}(\tan \varphi - \tan \varphi_{0} )] \hfill \\ \end{gathered} \right\},$$

and

19 $$\left. \begin{gathered} 0 \le z \le \frac{H}{2} - \frac{\Delta z}{2},\delta ^{\prime} = \tan^{ - 1} [\tan \delta_{0} + \frac{z}{H/2 - \Delta z/2}(\tan \delta - \tan \delta_{0} )] \hfill \\ \frac{H}{2} - \frac{\Delta z}{2} \le z \le \frac{H}{2} + \frac{\Delta z}{2},\delta ^{\prime} = \delta \hfill \\ \frac{H}{2} + \frac{\Delta z}{2} \le z \le H,\delta ^{\prime} = \tan^{ - 1} [\tan \delta_{0} + \frac{H - z}{{H/2 - \Delta z/2}}(\tan \delta - \tan \delta_{0} )] \hfill \\ \end{gathered} \right\},$$

where,

20 $$\varphi_{0} = \sin^{ - 1} \left[ {\frac{{1 - K_{0} }}{{1 + K_{0} }}} \right].$$

In which,

21 $$K_{0} = 1 - \sin \varphi .$$

**Figure 4:**
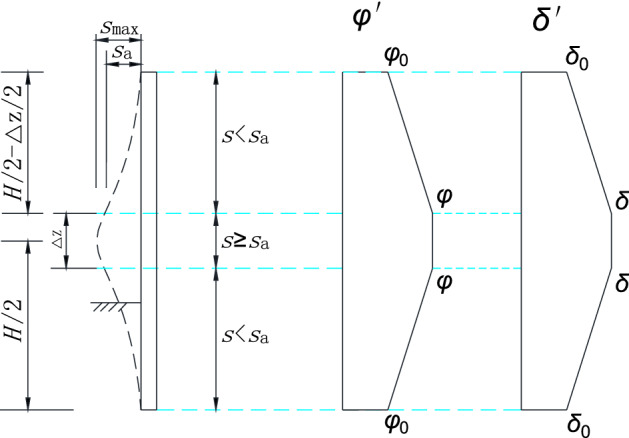
*φ*′ and *δ*′ along the height of retaining wall.

In general, *δ* = 2*φ*/3 and *δ*_0_ = *φ*/2. The variation of *φ*′ and *δ*′ along the retaining wall under drum deformation mode is shown in Fig. 4.

**Figure 5:**
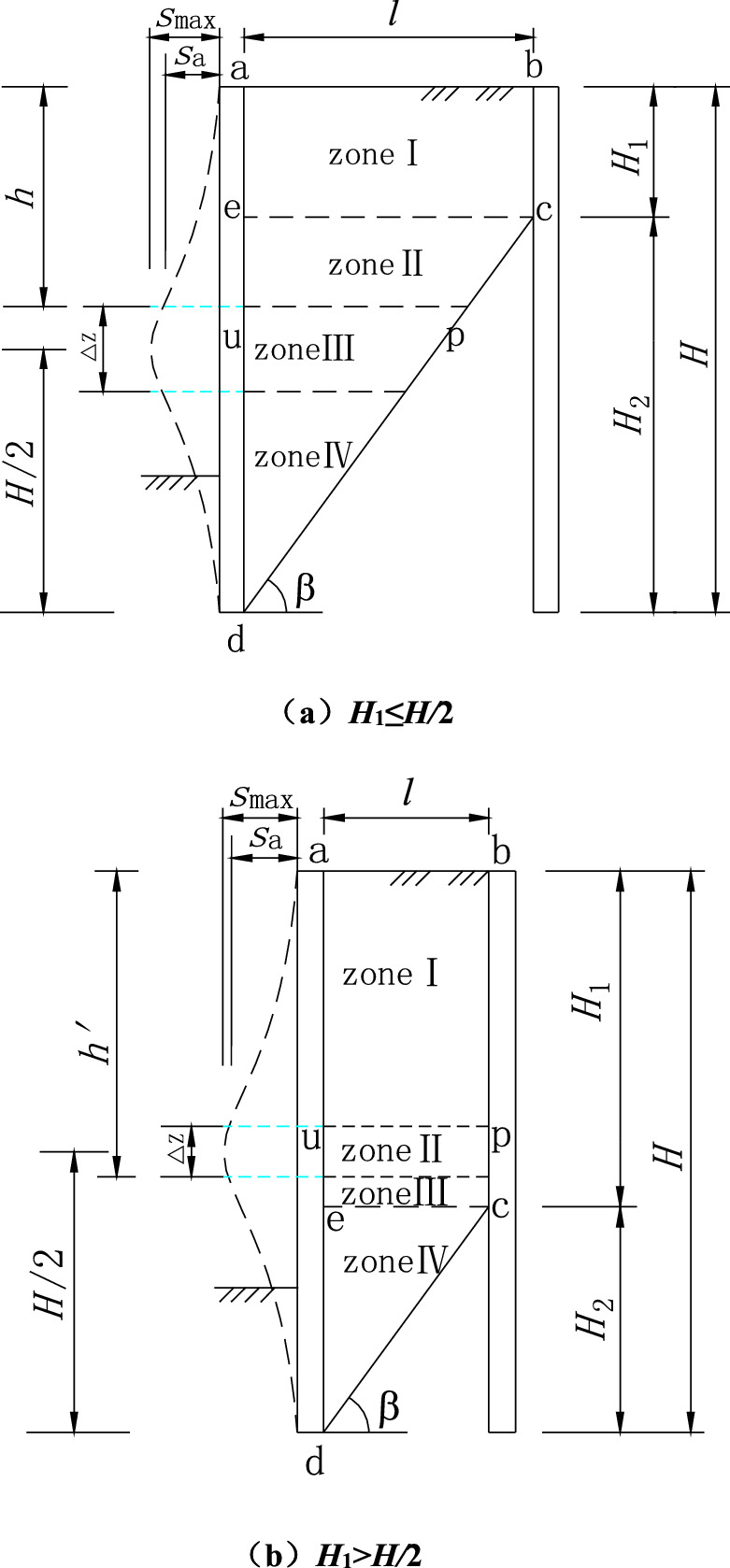
Drum deformation displacement mode of flexible retaining wall.

The height of the intermediate transition zone is set as *∆z* = *x*·*H*, the soil mass within the height *∆z* reaches the active limit equilibrium state. x is the ratio of soil layer height entering the limit state along the retaining wall. For the first case of *H*_1_ ≤ *H/*2, as shown in Fig. 5a, the depth from the top surface of the transition zone to the fill’s top surface is *h* = *H*/2 − *∆z*/2. This paper limits the depth *h* to [*H*_1_, *H*/2], where *h* tends to *H*_1_with *∆z* increases. When *∆z*/2 ≥ *H*/2 − *H*_1_, the original zone II disappears, and the depth of the top surface of the transition zone is calculated as *h* = *H*_1_. When *∆z* → 0, *h* tends to *H*/2 and is calculated as *h* = *H*/2, the thin transition layer shown in Fig. 1a.

For the second case of *H*_1_ > *H*/2, as shown in Fig. 5b, the height from the bottom of the transition zone to the top of the fill is set as *h*′ = *H*/2 + *∆z*/2. In this paper, the depth *h*′ is limited to [*H*/2, *H*_1_], where *h*′ tends to *H*_1_ with ∆z increases. When *∆z*/2 ≥ *H*_1_ − *H*/2, the original area III disappears, and the depth of the bottom surface of the transition zone is calculated as *h*′ = *H*_1_. When *∆z* → 0, *h*′ tends to *H*/2 and is calculated as *h*′ = *H*/2, which is the thin transition layer shown in Fig. 1b.

## Solution for active earth pressure

The shearing stress on the level unit surface usually is not considered under the translation mode (T) because the soil mass moves as a whole, and there is no relative movement between the horizontal soil layers. However, under the drum movement mode, the flexible retaining wall can be regarded as the upper retaining wall rotating about the top (RT) and the lower retaining wall rotating about the bottom of the wall (RB). Each soil layer produces relative motion in the rotation direction with respect to the below layer. Therefore, there must be level shearing stress between the upper and lower soil. The distribution of shearing stress is very complex, and it will affect the moment balance condition. If the moment equilibrium condition is not involved in the derivation, then the specific distribution of shear stress is not concerned^29,35^. Nevertheless, different stress distribution assumptions on the horizontal plane will not affect two static equilibrium of force along with the horizontal and vertical directions.

In this paper, the differential level layer method is introduced. According to the relative movement trend of the horizontal layer unit of the soil wedge behind the wall, the action direction of the friction shearing stress between the level layer units in each zone is determined^16,25,35,36^. Under the condition of satisfying the balance of forces, the active earth pressure differential equation in a non-limit state is established, and then its distribution is discussed.

### Zone I

**Figure 6:**
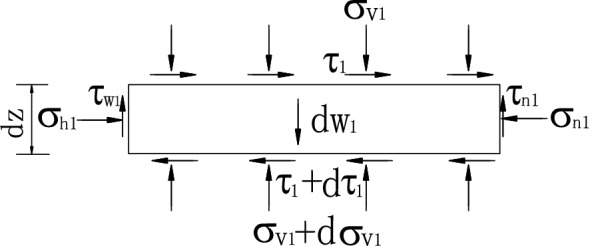
Forces acting on level units in zone I.

The level layer unit in zone I is shown in Fig. 6, and the static equilibrium equations are established.

22 $$\sigma_{h1} dz - \sigma_{n1} dz - nHd\tau_{1} = 0,$$

23 $$\tau_{w1} dz + nHd\sigma_{v1} + \tau_{n1} dz - dw_{1} = 0.$$

In which, the second order differentiation has been omitted, *σ*_v1_ is the vertical stress on the layer unit at depth *z*, and *σ*_h1_ is the lateral active earth pressure.

24 $$\sigma_{h1} = K_{awn1} \sigma_{v1} .$$

*τ*_1_ is the horizontal shearing stress on the surface of the layer unit, assuming it is average distribution, it can be obtained.

25 $$\tau_{1} = k_{1} \sigma_{v1} ,$$

where *τ*_w1_ is the shearing stress on the interface, and the magnitude of *τ*_w1_ is:

26 $$\tau_{w1} = \sigma_{h1} \tan \delta^{\prime}.$$

*σ*_n1_ is the horizontal lateral pressure on the outer wall at depth *z*, and *τ*n1 is the shearing stress. *τ*_n1_ can be expressed by

27 $$\tau_{n1} = \sigma_{n1} \tan \delta^{\prime}.$$

*dw*_1_ is the self-weight of the level unit in zone I, and its magnitude is obtained as:

28 $$dw_{1} = \gamma nHdz.$$

In general, there are

29 $$(1 - k_{1} \tan \delta^{\prime})\frac{{d\sigma_{v1} }}{dz} + \frac{{2K_{awn1} \tan \delta^{\prime}}}{nH}\sigma_{v1} - \gamma = 0.$$

When *z* = 0, *σ*_v1_ = 0 is regarded as the boundary condition of zone I, the first-order linear differential equation (Eq. 29) can be solved as follows.

30 $$\sigma_{v1} = \frac{\gamma }{B} - \frac{\gamma }{B}e^{{\left( { - \frac{B}{A}z} \right)}} .$$

In which

31 $$\left. \begin{gathered} A = 1 - k_{1} \tan \delta^{\prime} \hfill \\ B = \frac{{2K_{awn1} \tan \delta^{\prime}}}{nH} \hfill \\ \end{gathered} \right\}.$$

When *z* = *H*_1_, *σ*_v1_ = *D*_1_ can be regarded as the boundary condition of equivalent load on the surface of the isolated body in zone II.

32 $$D_{1} = \frac{\gamma }{B} - \frac{\gamma }{B}e^{{\left( { - \frac{B}{A}H_{1} } \right)}} .$$

### Zone II

**Figure 7:**
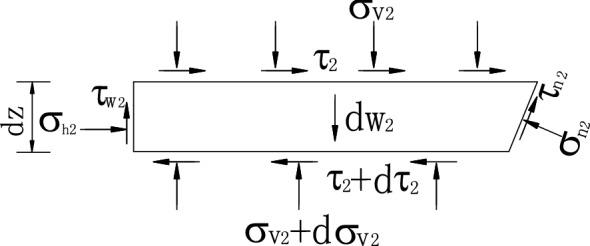
Forces acting on level units in zone II.

On the basis of the static equilibrium conditions of horizontal and vertical directions acting on the layer unit (Fig. 7), the equation is established. The second-order differential components are omitted to obtain.

33 $$\sigma_{h2} dz + \tau_{2} dz\cot \beta - d\tau_{2} (H - z)\cot \beta - \sigma_{n2} dz + \tau_{n2} \cot \beta dz = 0,$$

34 $$\tau_{w2} dz - \cot \beta \sigma_{v2} dz + (H - z)\cot \beta d\sigma_{v2} + \cot \beta \sigma_{n2} dz + \tau_{n2} dz - dw_{2} = 0,$$

where *σ*_v2_ is the mean vertical normal stress on the surface of layer unit, and *σ*_h2_ is the lateral active earth pressure.

35 $$\sigma_{h2} = K_{awn2} \sigma_{v2} .$$

*τ*_2_ is the level shearing stress on the soil layer unit. It is assumed to be uniformly distributed, and its magnitude is expressed as follows:

36 $$\tau_{2} = k_{2} \sigma_{v2} ,$$

where *τ*_w2_ refers to the shearing stress on the interface and its expression is

37 $$\tau_{w2} = \sigma_{h2} \tan \delta^{\prime}.$$

*σ*_n2_ is the normal stress distributed uniformly on the rupture surface. *τ*_n2_ is the shearing stress distributed uniformly, the formula is

38 $$\tau_{n2} = \sigma_{n2} \tan \varphi^{\prime}.$$

In which *dw*_2_ is the self-weight of level layer element in zone II, and its expression is

39 $$dw_{2} = \gamma (H - z)\cot \beta dz.$$

By synthesizing the above formula, the first order differential equation is obtained,

40 $$F\frac{{d\sigma_{v2} }}{dz} + G\frac{{\sigma_{v2} }}{(H - z)} - \gamma = 0$$

in which,

41 $$\left. \begin{gathered} F = (1 + k_{2} C) \hfill \\ G = \tan \beta [K_{awn2} \tan \delta^{\prime} - \cot \beta - C(k_{2} \cot \beta + K_{awn2} )] \hfill \\ C = \frac{{\cot \beta + \tan \varphi^{\prime}}}{{\cot \beta \tan \varphi^{\prime} - 1}} \hfill \\ \end{gathered} \right\}.$$

Equations (40) and (32) are solved to present the following equation.

42 $$\sigma_{v2} = \frac{ - \gamma (H - z)}{{F - G}} + \left[ {D_{1} + \frac{{\gamma (H - H_{1} )}}{F - G}} \right]\left( {\frac{H - z}{{H - H{}_{1}}}} \right)^{\frac{G}{F}} .$$

By substituting Eq. (42) into Eq. (35), the horizontal active earth pressure in zone II is derived.

When *z* = *h*, *σ*_v2_ = *D*_2_ is regarded as the boundary condition of equivalent load on zone III.

43 $$D_{2} = \frac{ - \gamma (H - h)}{{F - G}} + \left[ {D_{1} + \frac{{\gamma (H - H_{1} )}}{F - G}} \right]\left( {\frac{H - h}{{H - H{}_{1}}}} \right)^{\frac{G}{F}} .$$

### Zone III

**Figure 8:**
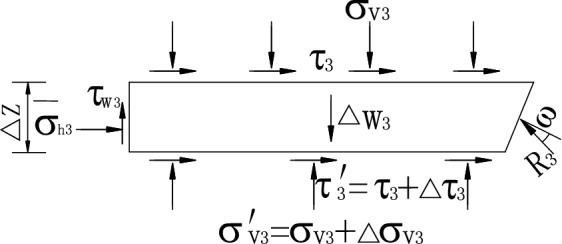
Forces acting on level units in zone III.

Zone III is the middle transition layer, in which the shearing strength of the soil is fully mobilized, the internal friction angle of fill is *φ*′ = *φ*, and the external friction angle between walls and soils is *δ*′ = *δ*. Therefore, the mean vertical compressive stress on the top of layer (*z* = *h*) is *σ*_v3_ = *D*_2_, and the mean vertical compressive stress at the bottom of the layer is *σ*′_v3_ = *σ*_v3_ + ∆*σ*_v3_, as shown in Fig. 8. When *∆z* is large, the whole isolator in zone III is taken as the research object, and the horizontal and vertical static balance equations are established as follows:

44 $$\overline{\sigma }_{{_{h3} }} \left( {\frac{\Delta z}{2} + \frac{H}{2} - h} \right) + k_{3} \sigma_{v3} (H - h)\cot \beta + k_{3} \sigma_{v3}^{^{\prime}} \left( {\frac{H}{2} - \frac{\Delta z}{2}} \right)\cot \beta - R_{3} \sin (\beta - \varphi ) = 0,$$

and

45 $$\overline{\sigma }_{{_{h3} }} \tan \delta \left( {\frac{\Delta z}{2} + \frac{H}{2} - h} \right) - \sigma_{v3} (H - h)\cot \beta + \sigma_{v3}^{^{\prime}} \left( {\frac{H}{2} - \frac{\Delta z}{2}} \right)\cot \beta - \Delta w_{3} + R_{3} \cos (\beta - \varphi ) = 0$$

in which, $\overline{\sigma }_{{_{h3} }}$ is the mean lateral horizontal stress.

46 $$\overline{\sigma }_{{_{h3} }} = k_{awn3} \left( {\frac{{\sigma_{v3} + \sigma_{v3}^{^{\prime}} }}{2}} \right).$$

Thus,

47 $$Q\sigma_{v3} + S\sigma_{v3}^{^{\prime}} - T = 0$$

in which,

48 $$\left. \begin{gathered} Q = (H - h)\cot \beta [k_{3} \cos (\beta - \varphi ) - \sin (\beta - \varphi )] + \frac{1}{2}k_{awn3} \left( {\frac{H}{2} + \frac{\Delta z}{2} - h} \right)[\cos (\beta - \varphi ) + \tan \delta \sin (\beta - \varphi )] \hfill \\ S = \left( {\frac{H}{2} - \frac{\Delta z}{2}} \right)\cot \beta [k_{3} \cos (\beta - \varphi ) + \sin (\beta - \varphi )] + \frac{1}{2}k_{awn3} \left( {\frac{H}{2} + \frac{\Delta z}{2} - h} \right)[\cos (\beta - \varphi ) + \tan \delta \sin (\beta - \varphi )] \hfill \\ T = \frac{1}{2}\gamma \cot \beta \sin (\beta - \varphi )\left( {\frac{3H}{2} - h - \frac{\Delta z}{2}} \right)\left( {\frac{H}{2} + \frac{\Delta z}{2} - h} \right) \hfill \\ \end{gathered} \right\}.$$

When *z* = *h*, *σ*_v3_ = *D*_2_ is substituted into Eq. (47) as a known loading condition, *σ*′_v3_ at the bottom of the layer can be obtained. Assuming *σ*′_v3_ = *D*_3_, it can also be regarded as the equivalent load on the insulator’s top surface in zone IV. The distribution of *σ*_h3_ along the height *∆z* of the middle transition zone can be approximately considered as a linear distribution, and its expression is

49 $$\sigma_{h3} = k_{awn3} \left[ {D_{2} + (D_{2} - D_{3} )\frac{h - z}{{(H/2 + \Delta z/2 - h)}}} \right].$$

When *∆z* → 0, *h* = *H*/2 is taken for calculation, and zone III is a thin transition layer. In accordance with its equilibrium conditions, the solution can be obtained.

50 $$\Delta \sigma_{{{\text{v3}}}} =\frac{{ - 2k_{3} \sigma_{{{\text{v3}}}} }}{{k_{3} + \tan (\beta - \varphi )}}.$$

The mean vertical stress at the bottom of the thin transition level unit is

51 $$\left. {\sigma_{v3}^{^{\prime}} } \right|_{z = h} = \sigma_{{{\text{v3}}}} + \Delta \sigma_{{{\text{v3}}}} =\frac{{ - k_{3} + \tan (\beta - \varphi )}}{{k_{3} + \tan (\beta - \varphi )}}\sigma_{{{\text{v3}}}} .$$

Therefore, when *∆z* → 0, the mean pressure stress at the bottom of the thin transition layer is taken as the equivalent load on the insulator’s top surface in zone IV, and the formula is as follows.

52 $$\left. {D_{3} } \right|_{z = h} =\frac{{ - k_{3} + \tan (\beta - \varphi )}}{{k_{3} + \tan (\beta - \varphi )}}D_{2} .$$

### Zone IV

**Figure 9:**
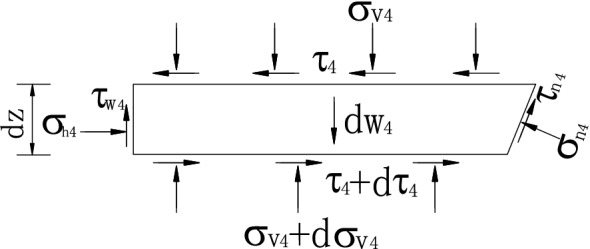
Forces acting on level units in zone IV.

From the static equilibrium conditions of the unit in horizontal and vertical directions (in Fig. 9), we can get:

53 $$\sigma_{h4} dz - \tau_{4} dz\cot \beta + d\tau_{4} (H - z)\cot \beta - \sigma_{n4} dz + \tau_{n4} \cot \beta dz = 0,$$

54 $$\tau_{w4} dz - \sigma_{v4} dz\cot \beta + (H - z)\cot \beta d\sigma_{v4} + \sigma_{n4} dz\cot \beta + \tau_{n4} dz - dw_{4} = 0.$$

In which, *σ*_v4_ is the mean direct stress on the layer unit’s surface at depth *z*, and*σ*_h4_ is the horizontal active earth pressure.

55 $$\sigma_{h4} = K_{awn4} \sigma_{v4} .$$

*τ*_4_ is the level shearing stress on the surface of the layer unit, assuming a mean distribution, and its expression is:

56 $$\tau_{4} = k_{4} \sigma_{v4} ,$$

where *τ*_w4_ is the shearing stress on the contact surface, and its expression is:

57 $$\tau_{w4} = \sigma_{h4} \tan \delta^{\prime}.$$

*σ*_n4_ is the normal stress and *τ*_n4_ is the friction shearing stress, which is expressed as:

58 $$\tau_{n4} = \sigma_{n4} \tan \varphi^{\prime}.$$

In which, *dw*_4_ is the self-weight of level layer unit in zone IV, which can be expressed as:

59 $$dw_{4} = \gamma (H - z)\cot \beta dz.$$

By synthesizing the above formula, the first order differential equation can be get,

60 $$J\frac{{d\sigma_{v4} }}{dz} + L\frac{{\sigma_{v4} }}{(H - z)} - \gamma = 0$$

in which,

61 $$\left. \begin{gathered} J = (1 - k_{4} C) \hfill \\ L = \tan \beta [K_{awn4} \tan \delta ^{\prime} - \cot \beta + C(k_{4} \cot \beta - K_{awn4} )] \hfill \\ C = \frac{\cot \beta + \tan \varphi ^{\prime}}{{\cot \beta \tan \varphi ^{\prime} - 1}} \hfill \\ \end{gathered} \right\}.$$

By using the boundary condition, i.e. the mean vertical stress *σ*_v4_ = *D*_3_ on the top of zone IV, we can solve the differential Eq. (60) and get

62 $$\sigma_{v4} = \frac{ - \gamma (H - z)}{{J - L}} + \left[ {D_{3} + \frac{\gamma (H/2 - \Delta z/2)}{{J - L}}} \right]\left( {\frac{H - z}{{H/2 - \Delta z/2}}} \right)^{\frac{L}{J}} .$$

By substituting the above Eq. (62) with Eq. (55), the lateral active earth pressure in zone IV is generated.

For calculating the second case of *H*_1_ > *H*/2, the same method can be used for analysis. Given the length of the paper, a detailed derivation is omitted. When *z* = 0, *σ*_v1_ = 0 is the boundary condition of zone I, and the vertical stress on the surface of the level unit at depth *z* in zone I is obtained as:

63 $$\sigma_{v1} = \frac{\gamma }{B} - \frac{\gamma }{B}e^{{\left( { - \frac{B}{A}z} \right)}} .$$

When *z* = *H*/2 − *∆z*/2, *σ*_v1_ = *D*_1_ is regarded as the equivalent load on the top surface of zone II.

64 $$D_{1} = \frac{\gamma }{B} - \frac{\gamma }{B}e^{{ - \frac{B}{A}\left( {\frac{H}{2} - \frac{\Delta z}{2}} \right)}} .$$

Taking into account the overall static balance of the middle transition layer in zone II, we can get

65 $$Q^{\prime}\sigma_{v2} + S^{\prime}\sigma_{v2}^{^{\prime}} - T^{\prime} = 0$$

in which,

66 $$\left. \begin{gathered} Q^{\prime} = k_{awn2} \tan \delta \left( {\frac{\Delta z}{2} + h - \frac{H}{2}} \right) + (k_{2} \tan \delta - 1)nH \hfill \\ S^{\prime} = k_{awn2} \tan \delta \left( {\frac{\Delta z}{2} + h - \frac{H}{2}} \right) + (k_{2} \tan \delta + 1)nH \hfill \\ T^{\prime} = \gamma nH\left( {\frac{\Delta z}{2} + h - \frac{H}{2}} \right) \hfill \\ \end{gathered} \right\}.$$

Taking *σ*_v2_ = *D*_1_ at depth *z* = *H*/2 − *∆z*/2 as the known loading conditions, *σ*′_v2_ at the bottom of the layer can be obtained. Assuming *σ*′_v2_ = *D*_2_, it can also be regarded as the equivalent load on the top surface of zone III.

Similarly, the distribution of the earth pressure *σ*_h2_ along the height *∆z* of the middle transition zone can be considered a linear distribution approximately.

67 $$\sigma_{h2} = k_{awn2} \left[ {D_{1} + (D_{1} - D_{2} )\frac{H/2 - \Delta z/2 - z}{{(\Delta z/2 + h - H/2)}}} \right].$$

Considering the equivalent load on the top surface of zone III, the vertical stress on the surface of the level unit at depth *z* is

68 $$\sigma_{v3} = \frac{\gamma }{{G^{\prime}}} + \left( {D_{2} - \frac{\gamma }{{G^{\prime}}}} \right)e^{{\frac{{G^{\prime}}}{{F^{\prime}}}(h - z)}}$$

in which,

69 $$\left. \begin{gathered} F^{\prime} = 1 + k_{3} \tan \delta^{\prime} \hfill \\ G^{\prime} = \frac{{2K_{awn3} \tan \delta^{\prime}}}{nH} \hfill \\ \end{gathered} \right\}.$$

Then we can take *σ*_v3_ = *D*_3_ at depth *z* = *H*_1_ as the known loading conditions of equivalent load on the top surface of zone IV.

70 $$D_{3} = \frac{\gamma }{{G^{\prime}}} + \left( {D_{2} - \frac{\gamma }{{G^{\prime}}}} \right)e^{{\frac{{G^{\prime}}}{{F^{\prime}}}(h - H_{1} )}} .$$

The vertical stress in zone IV is

71 $$\sigma_{v4} = \frac{ - \gamma (H - z)}{{J - L}} + \left( {D_{3} + \frac{{\gamma H_{2} }}{J - L}} \right)\left( {\frac{H - z}{{H_{2} }}} \right)^{\frac{L}{J}} .$$

## Resultant force and height of action point

When *σ*_h1_, *σ*_h2_, *σ*_h3_, and *σ*_h4_ are integrated along with the wall height, the horizontal component *E*_ax_ of resultant force for earth pressure on the whole retaining wall can be obtained.

72 $$\begin{aligned} E_{ax} & = \int_{0}^{{H_{1} }} {\sigma_{h1} } dz + \int_{{H_{1} }}^{h} {\sigma_{h2} dz + \int_{h}^{(H/2 + \Delta z/2} {\sigma_{h3} } dz\int_{(H/2 + \Delta z/2)}^{H} {\sigma_{h4} } dz} \\ & = \gamma K_{awn1} \left[ {\frac{{H_{1} }}{B} + \frac{A}{{B^{2} }}[e^{{\left( { - \frac{B}{A}H_{1} } \right)}} - 1]} \right] + \frac{{\gamma K_{awn2} }}{F - G}\left( {\frac{{h^{2} }}{2} - Hh - \frac{{H_{1}^{2} }}{2} + HH_{1} } \right) \\ & \quad + \frac{{FK_{awn2} }}{F + G}\left( {D_{1} + \frac{{\gamma H_{2} }}{F - G}} \right)\left[ {(h - H)\left( {\frac{H - h}{{H_{2} }}} \right)^{\frac{G}{F}} + H_{2} } \right] + K_{awn3} \left( {\frac{{D{}_{2} + D_{3} }}{2}} \right)\left( {\frac{H}{2} + \frac{\Delta z}{2} - h} \right) \\ & \quad + \frac{{K_{awn4} (H - \Delta z)}}{8(J + L)}[\gamma (H - \Delta z) + 4JD_{3} ]. \\ \end{aligned}$$

The expression of resultant force is given by

73 $$E_{a} = \frac{{E_{ax} }}{\cos \delta }.$$

The height *y* of the point of application of the resultant force is as follows

74 $$\begin{aligned} y & = \frac{{\int_{0}^{{H_{1} }} {\sigma_{h1} (H - z)} dz + \int_{{H_{1} }}^{h} {\sigma_{h2} (H - z)dz + \int_{h}^{(H/2 + \Delta z/2)} {\sigma_{h3} (H - z)dz} + \int_{(H/2 + \Delta z/2)}^{H} {\sigma_{h4} (H - z)dz} } }}{{E_{ax} }} \\ & = \frac{1}{{E_{ax} }} \cdot \left[ \begin{gathered} \frac{{K_{awn1} \gamma }}{B}\left( { - \frac{{H_{1}^{2} }}{2} + HH_{1} - \frac{AH}{B} + \frac{{A^{2} }}{{B^{2} }}} \right) + \frac{A}{{B^{2} }}K_{awn1} \gamma e^{{\left( { - \frac{{BH_{1} }}{A}} \right)}} \left( {H_{2} - \frac{A}{B}} \right) \hfill \\ + \frac{{K_{awn2} \gamma }}{3(F - G)}\left[ {(H - h)^{3} - H_{2}^{3} } \right] - \frac{{FK_{awn2} }}{G + 2F}\left( {D_{1} + \frac{{\gamma H_{2} }}{F - G}} \right)\left[ {(h - H)^{2} \left( {\frac{H - h}{{H_{2} }}} \right)^{\frac{G}{F}} - H_{2}^{2} } \right] \hfill \\ + \frac{{K_{awn3} }}{4}(D_{2} + D_{3} )\left( {\frac{H}{2} + \frac{\Delta z}{2} - h} \right)\left( {\frac{3H}{2} - h - \frac{\Delta z}{2}} \right) \hfill \\ + \frac{{K_{awn4} (H - \Delta z)^{2} [\gamma (H - \Delta z) + 6JD_{3} ]}}{24(L + 2J)} \hfill \\ \end{gathered} \right]. \\ \end{aligned}$$

In the first case, when *∆z*/2 ≥ *H*/2 − *H*_1_, the depth of the top surface of the transition zone is *h* = *H*_1_, and the calculation height [*H*_1_, *h*] of zone II is zero. In the calculation formula of *E*_ax_ and *y*, the calculation components in zone II are zero, and the original zone II is canceled. For the second case, when *∆z*/2 ≥ *H*_1_ − *H*/2, the calculated height of zone III is zero, and zone III is actually canceled.

When the aspect ratio *n* is large, the slip plane slides out from the soil’s top surface. In this paper, *H*_1_ = 0, *σ*_h1_ in zone I is always zero, and the calculation components of zone I in the calculation formula of *E*_ax_ and *y* are all zero. In fact, the original zone I has been canceled, and this problem has developed into the earth pressure problem of infinite soil.

## Model test

**Figure 10:**
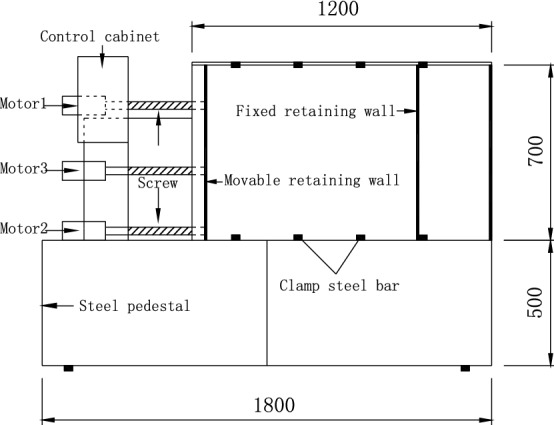
Construction detail of test box(unit: mm).

**Figure 11:**
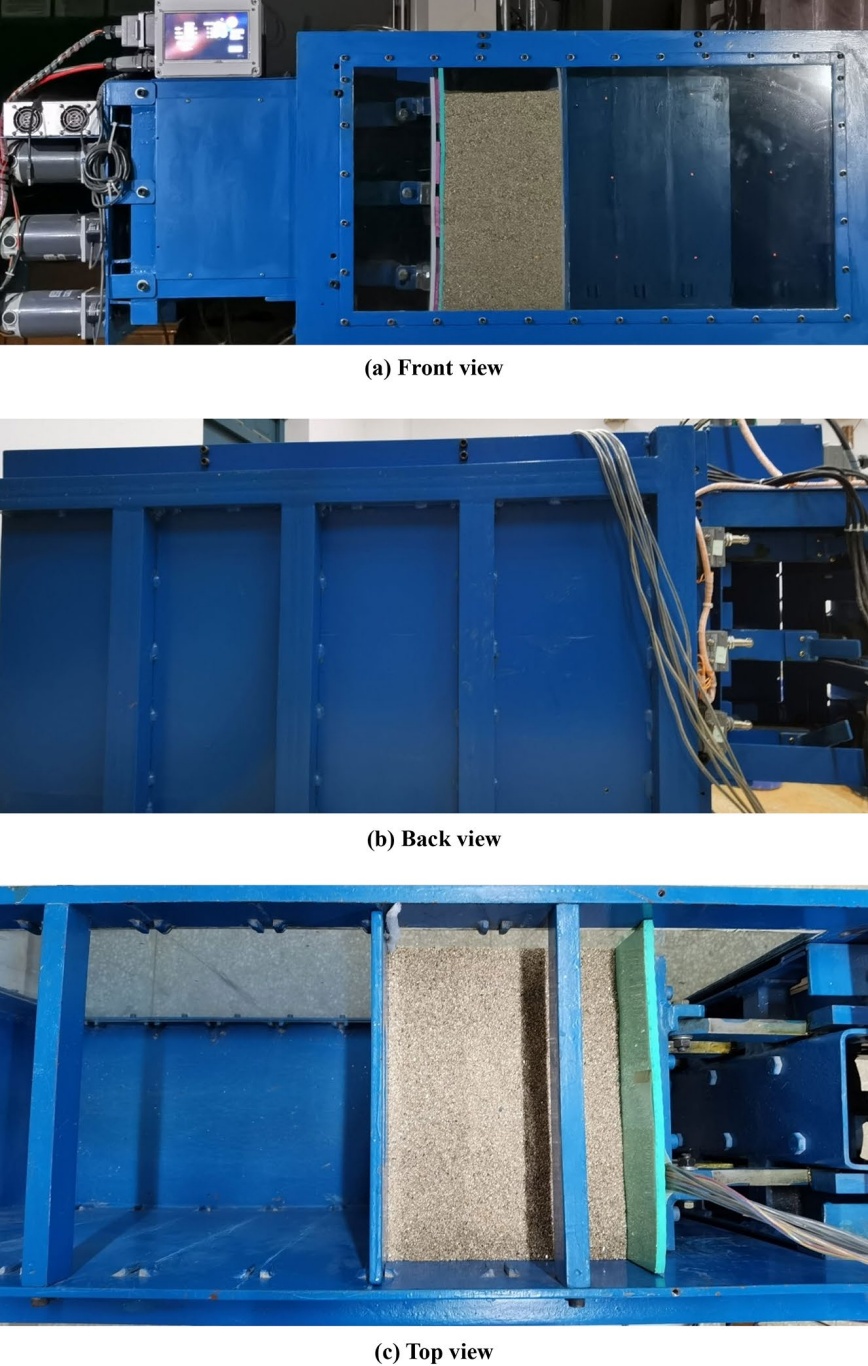
Entity of test box.

As shown in Fig. 10, the self-made model is used for the experimental study^37–39^. The movable baffle on the left side of the sandbox is polypropylene plate, the fixed baffle on the right side is steel plate, the front side baffle is tempered glass, and the backside baffle is frame steel plate, simulating the flexible retaining wall close to the outer wall of the basement. The real object of the test model device is shown in Fig. 11.

**Figure 12:**
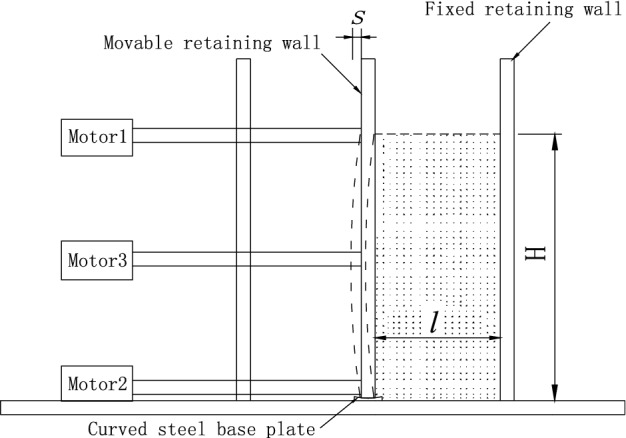
Drum deformation mode of flexible retaining wall.

Three motors are installed on the outer side of the sand loading box, as shown in Fig. 12. During the test, the upper and lower motors do not operate (simulating that the top of the retaining wall is supported and the bottom of the retaining wall is embedded). When the middle motor is running, the transmission shaft rotates slowly. The center of the movable baffle gradually moves horizontally outward, forming a certain horizontal displacement and realizing the drum-shaped deformation displacement mode. Taking the soil with limited width in the test box as the PIV analysis area, the digital camera is used to take photos automatically, and the shooting time interval is 1–2 s. Two light sources are placed on both sides of the sandbox to reduce specular reflection. Finally, the images are processed by PIV analysis software.

**Figure 13:**
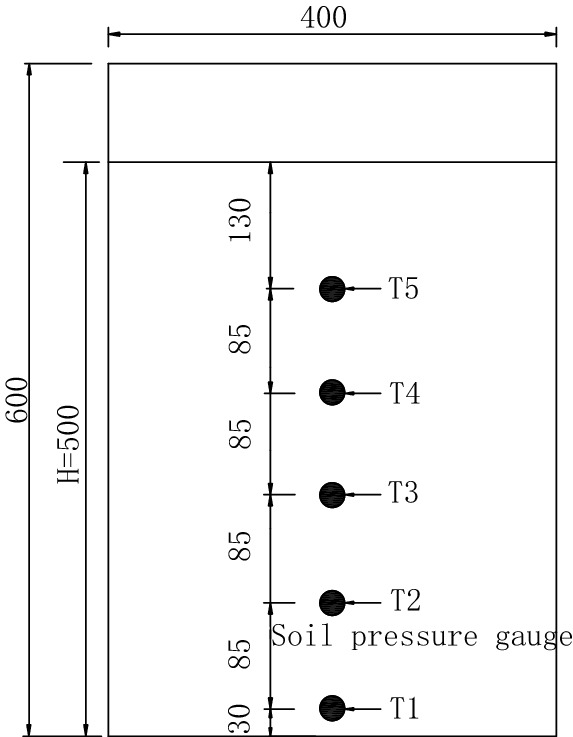
Layout of earth pressure cells (unit: mm).

The earth pressure is measured by five CYY9 micro earth pressure gauges arranged on the movable retaining wall, with a measuring range of 5 kPa and a size of Ф 22 mm × 13 mm. First, the groove is excavated along the vertical centerline of the movable baffle at different depths, and the micro-earth pressure gauges are embedded. The groove’s depth is the same as the gauge’s thickness to reduce the influence of the gauges protruding from the baffle. Then, a hole is drilled at the groove side to lead out the wire of the earth pressure gauge from the back of the wall, as shown in Fig. 13.

Four groups of soil pressure tests were carried out in this model test, and the specific test parameters are shown in Table 1. The mechanical parameters of sand samples are cohesion *c* = 0, internal friction angle *φ* = 36.5°, wall friction angle *δ* = 24.3°, and unit weight of the tested sand specimen *γ* = 15 kN/m^3^.

**Table 1:** Parameters of tests.

Number	Height/m	Width/m	Ratio of width to height
1^#^	0.50	0.10	0.2
2^#^	0.50	0.15	0.3
3^#^	0.50	0.20	0.4
4^#^	0.50	0.25	0.5

### Soil deformation analysis

**Figure 14:**
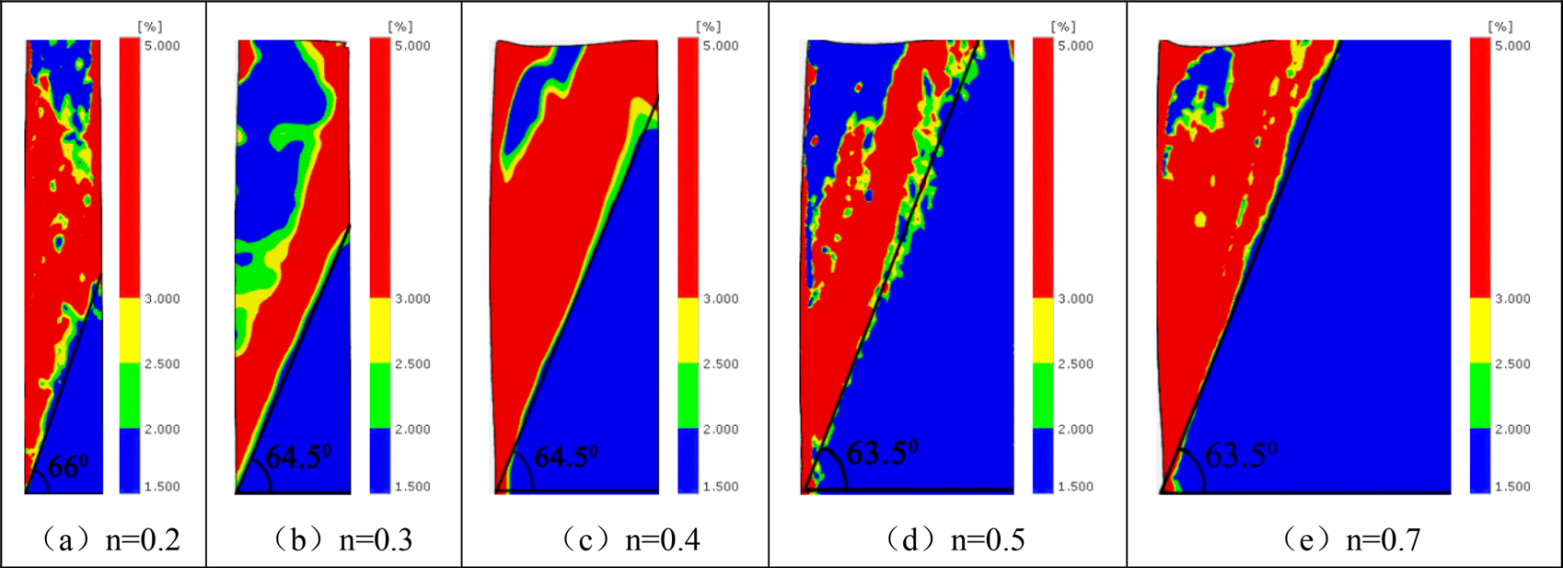
Displacement fields of soil for different n (H = 500 mm).

Taking the limited width as the PIV analysis area, image processing is carried out^40,41^. The deformation displacement diagram of different width sand (*n* = 0.2, 0.3, 0.4, 0.5, 0.7) under the drum movement mode is gained, as shown in Fig. 14.

As shown in Fig. 14, the sliding failure surface is a plane developing upward from the bottom of the movable baffle. With the increase of the filled sand’s width, the intersection of the slip plane and fixed baffle moves upward until the slip plane slides out from the filled sand’s top surface. When the ratio of width to height increases from 0.4 to 0.5, the intersection point of the slip plane gradually changes from fixed retaining wall to sand top surface. Therefore, it can be judged that the critical ratio of width height ratio of the finite soil in the model test is between 0.4 and 0.5. When the sand width is greater than the critical width, the retaining soil is considered semi-infinite.

The ultimate fracture surface inclination angle is measured and compared with that calculated on the basis of the generalized Coulomb method, as shown in Table 2. It can be seen that the model test results are close to the theoretical calculation results in the limited width range. With the increase of aspect ratio, the ultimate fracture angle decreases gradually and becomes stable. The experimental analysis shows that the fracture angle *β* approaches to π/4 + φ/2 = 63.25° under the infinite width (width height ratio *n* = 0.5, 0.7).

**Table 2:** Slip surface inclinations under different *n.*

Number	Ratio of width to height	Test result/(°)	Calculation result/(°)
1^#^	0.2	66.0	64.22
2^#^	0.3	64.5	63.20
3^#^	0.4	64.5	62.07
4^#^	0.5	63.5	60.86
5^#^	0.7	63.5	60.08

### Earth pressure test results

**Figure 15:**
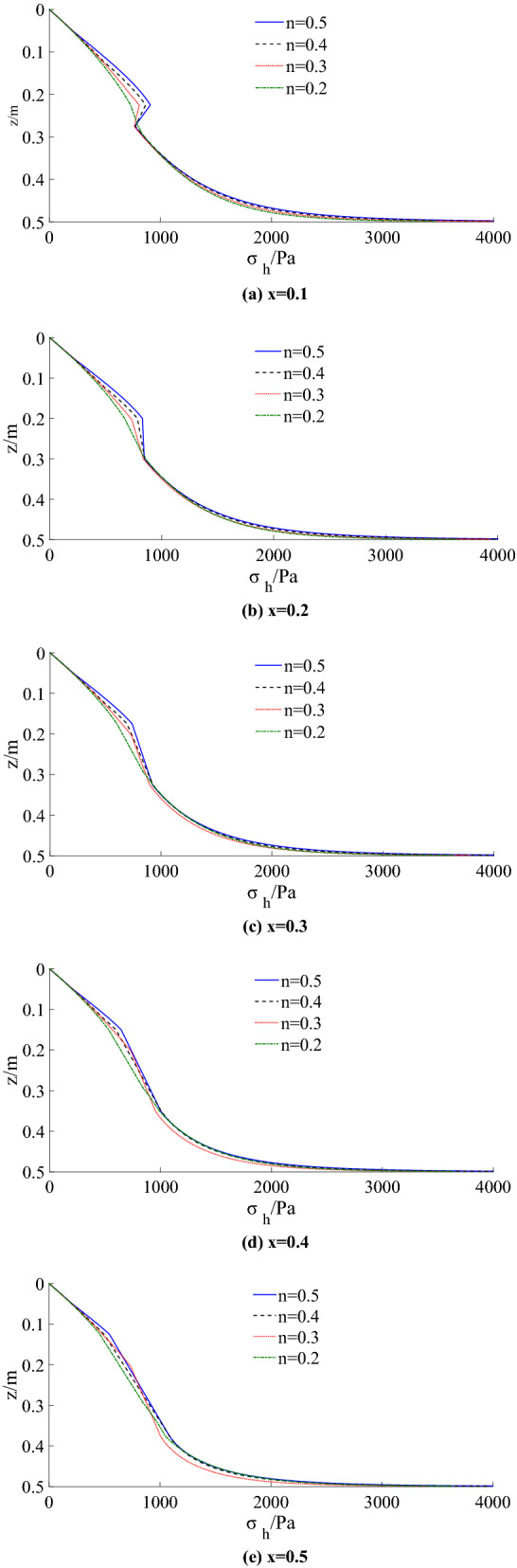
Theoretical calculation results under different x.

By using the theoretical method in this paper, the distribution of lateral active earth pressure with different ratios (*n* = 0.2, 0.3, 0.4, 0.5) in the model test is calculated, as shown in Fig. 15.

The theoretical calculation solution shows that the active earth pressure in the figure is nonlinear along with the height of the wall. The lateral earth pressure reduces with the reduction of ratio within the limited width. When the ratio decreases to *n* = 0.2, the lateral earth pressure decreases significantly. As the limit state region (*x* from 0.1 to 0.5) increases, the lateral earth pressure near the retaining wall’s top and bottom decreases gradually. In contrast, the lateral earth pressure in the middle of the retaining wall does not change significantly.

**Figure 16:**
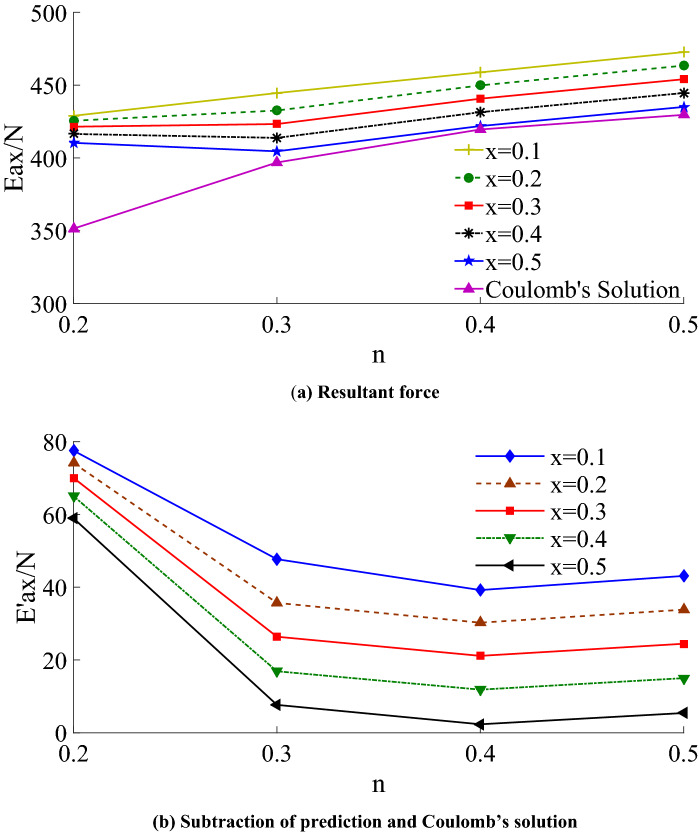
Comparison of theoretical solution and Coulomb’s solution.

The resultant force increases with the increase of width to height and decreases with the rise of limit state area under the same ratio. When the width (reaching and exceeding the critical width) and the limit state region increase, the resultant thrust approach to that of Coulomb’s result, which is consistent with the previous study, as shown in Fig. 16a. *E*'_ax_ is obtained by subtracting the prediction and Coulomb's solution. Figure 16b shows the difference at different x.

**Figure 17:**
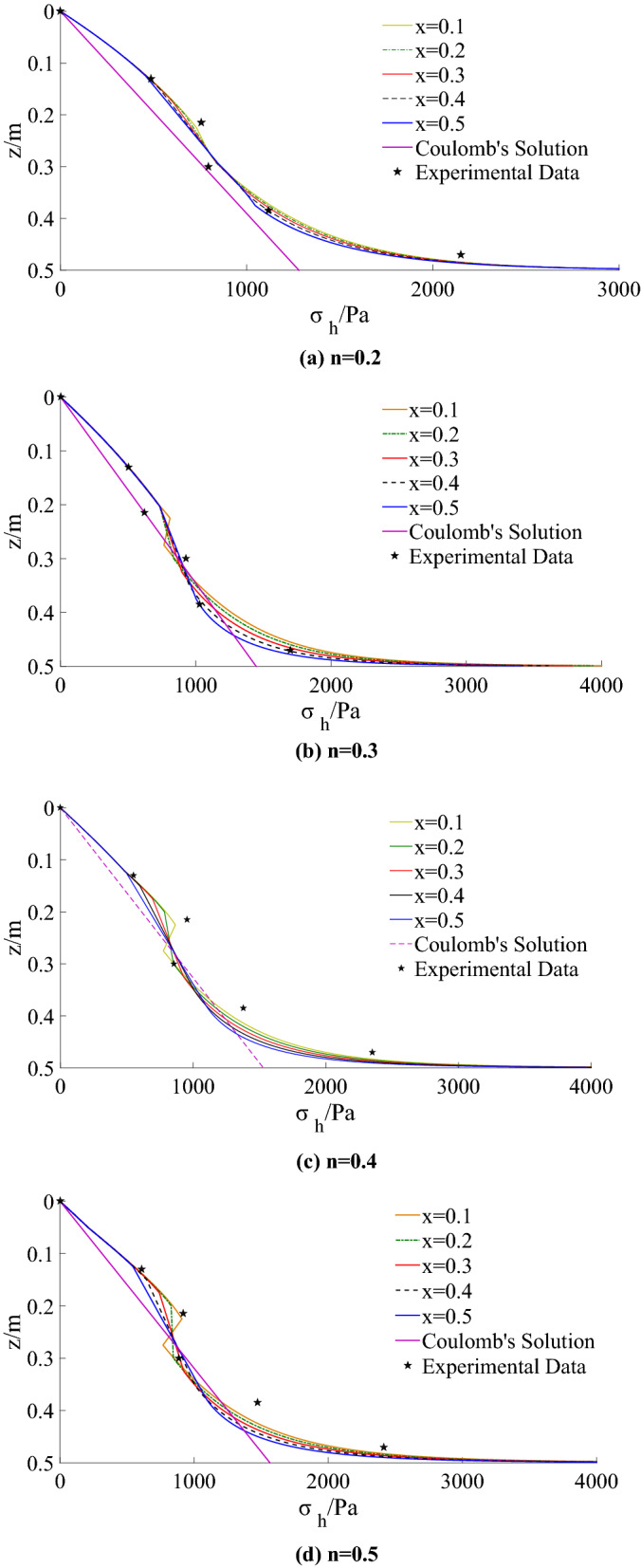
Comparison of theoretical calculation with experimental results.

Figure 17 and Table 3 show the comparison of the theoretical calculation of lateral earth pressure distribution with the test results. It can be seen from the figure that the initial horizontal displacement of the retaining wall is smaller under the same ratio, and the limit state region is also tiny, which is confined to the middle part of the wall. The lateral earth pressure on the upper and bottom of the wall is relatively large and highly nonlinear. With the increase of the drum deflection, most central areas enter the active limit state, and the horizontal displacement increases while the earth pressure decreases. With the increase of the limit state region (*x* from 0.1 to 0.5), the horizontal lateral earth pressure distribution tends to be linearized gradually, and it is close to Coulomb’s distribution of finite soil.

**Table 3:** Comparison of theoretical calculation with experimental results and Coulomb’s results.

n	Solution	Lateral earth pressure/Pa				
		z = 0.13 m	z = 0.215 m	z = 0.3 m	z = 0.385 m	z = 0.47 m
n = 0.2	Experiment Data	486.0	755.0	795.0	1118	2150
	Coulomb’s Solution	332.8	550.3	767.9	985.5	1203
	x = 0.5	469.2	665.2	864.3	1084	1743
	x = 0.4	475.4	673.3	861.3	1117	1782
	x = 0.3	477.5	683.3	858.8	1143	1810
	x = 0.2	479.1	696.3	856.7	1161	1830
	x = 0.1	480.4	708.7	857.3	1175	1845
n = 0.3	Experiment Data	504.0	620.0	926.0	1028	1700
	Coulomb’s Solution	376.1	622.0	867.9	1114	1360
	x = 0.5	499.0	752.9	885.9	1027	1561
	x = 0.4	500.5	752.0	878.5	1058	1669
	x = 0.3	501.9	750.2	864.0	1101	1766
	x = 0.2	502.9	746.9	837.3	1147	1852
	x = 0.1	503.7	778.2	844.0	1184	1918
n = 0.4	Experiment data	550.0	952.0	856.0	1379	2350
	Coulomb’s Solution	397.0	656.5	916.0	1175	1435
	x = 0.5	508.1	708.2	908.3	1110	1702
	x = 0.4	521.4	730.5	903.5	1115	1785
	x = 0.3	522.8	756.5	888.8	1140	1862
	x = 0.2	523.8	793.8	851.5	1172	1993
	x = 0.1	524.6	837.7	855.5	1206	1998
n = 0.5	Experiment data	610.0	917.0	884.0	1472	2415
	Coulomb’s Solution	403.6	667.5	931.4	1196	1459
	x = 0.5	555.1	738.6	922.1	1111	1749
	x = 0.4	570.6	761.4	912.9	1118	1835
	x = 0.3	573.5	789.2	892.4	1143	1914
	x = 0.2	575.7	831.2	845.3	1175	1987
	x = 0.1	577.2	885.3	849.5	1210	2055

The distribution of the earth pressure on the retaining wall calculated theoretically is consistent with that of the model test, and the distribution of the horizontal earth pressure in the middle area is concave. The drum deformation mode of retaining wall under different aspect ratios can be deemed that the upper part rotates about the top of the wall and the lower part rotates about the bottom of the wall. The supporting anchor structure restrains the upper part of the retaining wall, and the bottom is controlled by the fixed end, making the upper and bottom soil layers fail to reach the limit state completely. They are still in the active middle state, that is the non-limit active state, and the shearing strength of the soil is insufficient. Therefore, the earth pressure distribution of the upper and the bottom measuring points on the retaining wall are greater than Coulomb’s solution to the finite soil, while the middle area is entirely in the active limit state, and the earth pressure distribution of the intermediate measuring points are very close to the Coulomb’s solution to the finite soil.

**Figure 18:**
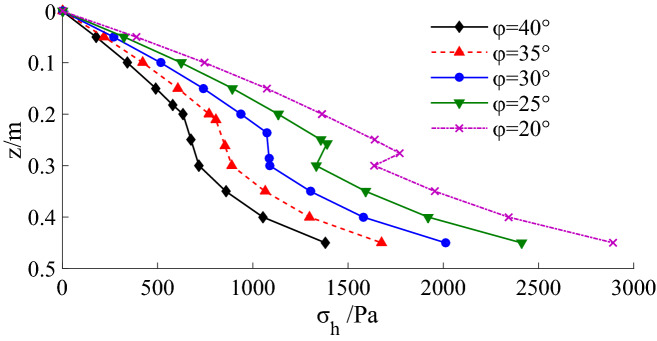
Active earth pressure distribution with different internal friction angle.

**Figure 19:**
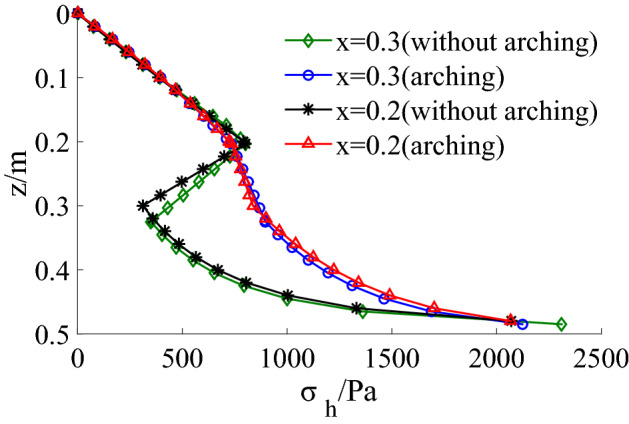
Theoretical calculation results with arching and without arching (n = 0.3).

The internal friction angle is an essential factor affecting the active earth pressure. When n = 0.3 and x = 0.2, the active earth pressure decreases with the increase of internal friction angle, as shown in Fig. 18. The active earth pressure without considering the soil arching is calculated and compared with the theoretical solution in this paper, as shown in Fig. 19. When the soil arching is not considered, the active earth pressure acting on the upper part of the retaining wall is almost the same as considering the soil arching. However, the earth pressure exerted on the middle and lower part of the retaining wall is obviously less than the earth pressure considering the soil arching. In particular, the earth pressure in the transition zone decreases obviously.

## Verification by comparison

**Figure 20:**
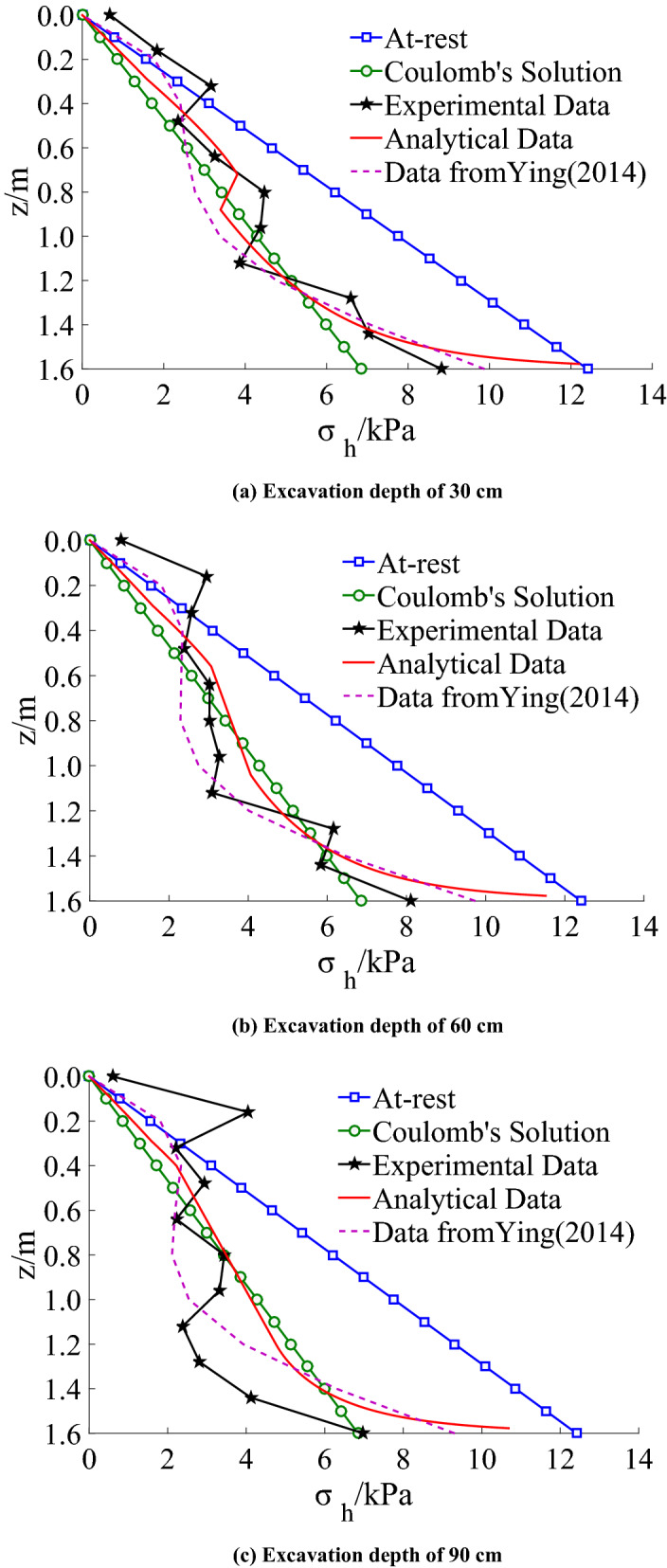
Distributions of horizontal earth pressures.

Taking Lu's model test^4^ as an example, dry sand is used in the test, *γ* = 16 kN/m^3^, *φ* = 31°, *δ* = 2*φ*/3, and the height of the flexible retaining wall with a single anchor is 2 m. Thus, the displacement of the wall under each excavation condition is typical drum deformation. By the theoretical method in this paper, the lateral earth pressure distribution is calculated for soils with infinite width (the ratio of width to height is taken as *n* = 0.5). The distribution of earth pressure at different excavation depths obtained by this method, Ying’s method^5^, and Lu's test^4^ is shown in Fig. 20.

The calculated results of the proposed method are close to the calculated results of Ying^5^ and the measured values of the model test by Lu^4^, and the earth pressure distribution law is the same. The results show that the earth pressure on the middle part of the retaining wall decreases with the drum deformation and the horizontal displacement, even less than the Coulomb earth pressure strength. On the other hand, the earth pressure strength on the upper and lower part of the retaining wall is greater than Coulomb’s solution due to soils being in a non-limit state.

## Conclusion

1. Based on the characteristics of the drum deformation mode of the flexible retaining wall close to the outer wall of the basement, four zones are divided to establish the mechanical analysis model for the solution of the active earth pressure. The analysis model takes account of the relative movement trend of the fill with the limited width.
2. The active earth pressure coefficient is obtained using the soil arch theory and considering the horizontal shearing stress between differential layers. Considering the drum deformation of the retaining wall and the non-limit state of upper and lower soil layers, the linear relationship between the mobilization of internal friction angle and external friction angle and the magnitude of displacement is presented, and the differential layer analysis method is modified.
3. The model tests are conducted, it is found that the failure angle reduces gradually and becomes stable with the increase of the ratio of width to height. When the ratio rises to infinite soil, the failure angle approaches *π*/4 + *φ*/2.
4. The test results show that the active earth pressure of soils with finite width is nonlinear, and the lateral earth pressure reduces with the reduction of the ratio of width to height in the critical width range. Furthermore, as the limit state region increases, the resultant force of earth pressure decreases under the same ratio of width to height.
5. The earth pressure strength on the upper and bottom parts of the retaining wall is greater than the Coulomb solution for finite soil. The earth pressure strength on the middle part of the retaining wall decreases continuously, less than the Coulomb earth pressure strength. As a result, the concave in the middle of the distribution curve is close to a linear line, and the lower part of the distribution curve has higher nonlinearity.

## Data Availability

All data, models, and code generated or used during the study appear in the submitted article.
